# Antifungal and Antioxidant Activities of Pyrrolidone Thiosemicarbazone Complexes

**DOI:** 10.1155/2012/795812

**Published:** 2012-02-01

**Authors:** Ahmed A. Al-Amiery, Abdul Amir H. Kadhum, Abu Bakar Mohamad

**Affiliations:** ^1^Department of Chemical and Process Engineering, Faculty of Engineering and Built Environment, University of Kebangsaan Malaysia, 43600 Bangi, Selangor, Malaysia; ^2^Biotechnology Division, Applied Science Department, University of Technology, Baghdad 10066, Iraq

## Abstract

Metal complexes of (Z)-2-(pyrrolidin-2-ylidene)hydrazinecarbothioamide (L) with Cu(II), Co(II), and Ni(II) chlorides were tested against selected types of fungi and were found to have significant antifungal activities. The free-radical-scavenging ability of the metal complexes was determined by their interaction with the stable free radical 2,2′′-diphenyl-1-picrylhydrazyl, and all the compounds showed encouraging antioxidant activities. DFT calculations of the Cu complex were performed using molecular structures with optimized geometries. Molecular orbital calculations provide a detailed description of the orbitals, including spatial characteristics, nodal patterns, and the contributions of individual atoms.

## 1. Introduction

Schiff bases have often been used as chelating ligands in the field of coordination chemistry, and their metal complexes have been of great interest to researchers for many years. It is well known that N and S atoms play a key role in the coordination of metals at the active sites of many metallobiomolecules [1]. The importance of metal ions in biological systems is well established. One of the most interesting features of metal-coordinated systems is the concerted spatial arrangement of the ligands around the metal ion. Among metal ions of biological importance, the Cu(II) ion involved in a large number of distorted complexes [2]. Over the past two decades, considerable attention has been paid to metal complexes of Schiff bases containing nitrogen and other donor atoms [3, 4]. Bioorganometallic chemistry is dedicated to the study of metallic complexes and their biological applications [5], including the design of new drugs that are more effective than those already known. The development of the field of bioinorganic chemistry has increased the interest in Schiff base complexes, because it has been recognized that many of these complexes may serve as models for biologically important species [6–9]. Antioxidants are extensively studied for their capacity to protect organisms and cells from damage induced by oxidative stress. Scientists in various disciplines have become more interested in new compounds, either synthesized or obtained from natural sources, that could provide active components to prevent or reduce the impact of oxidative stress on cells [10]. 

Thiosemicarbazones are well established as an important class of sulfur-donor Schiff base ligands that are particularly useful for transition metal ions. This is due to the remarkable biological activities observed for these compounds, which have been shown to be related to their metal-complexing ability. Thiosemicarbazone Schiff bases are an important class of compounds in the medicinal and pharmaceutical fields [11]. 

The work discussed herein describes the *in vitro* antioxidant and antifungal activities for metal complexes derived from (Z)-2-(pyrrolidin-2-ylidene)hydrazinecarbothioamide (L) [12].

## 2. Experimental

### 2.1. General

All chemicals used in this study were of reagent grade (supplied either by Sigma-Aldrich or Fluka) and used without further purification. 

The FTIR spectra were recorded in the 4.000–200 cm^−1^ range on cesium iodide windows using a Shimadzu FTIR 8300 spectrophotometer. Proton NMR spectra were recorded on a Bruker-DPX 300 MHz spectrometer using TMS as an internal standard. The UV-VIS spectra were measured in ethanol using the Shimadzu UV-VIS -160A spectrophotometer in the range 200–1.000 nm. Magnetic susceptibility measurements for the complexes were obtained at room temperature using a Magnetic Susceptibility Balance Model MSB-MKI. Flame atomic absorption spectra from the Shimadzu AA-670 elemental analyzer were used for metal determination. Elemental microanalysis was performed using a CHN elemental analyzer model 5500-Carlo Erba. A Gallenkamp M.F.B.600.010 F melting point apparatus was used to measure the melting points of all the synthesized compounds.

### 2.2. Chemistry

The ligand and metal complexes were synthesized according to reference [12], and the structures of the compounds were confirmed with elemental analyses, spectral analyses (IR, UV-VIS, ^1^H-NMR), conductance experiments, and magnetic measurements.

#### 2.2.1. DFT

The molecular sketches of the reference compounds were plotted using Visualization Materials Studio 5.5 software. All quantum chemical calculations were performed using density functional theory (DFT) methodology. The DMol3 model was employed to obtain quantum chemical parameters and optimization of the molecular geometry. Molecular atomic charges were calculated by Mulliken population analysis [13].

### 2.3. Pharmacology

#### 2.3.1. Evaluation of Antifungal Assay

All tests with the microorganisms (*Aspergillus niger* and *Candida albicans*) were obtained from the Biotechnology Division, Department of Applied Science, University of Technology.

Antifungal activity [14–16] was determined based on the growth inhibition rates of the mycelia of *Aspergillus niger* and *Candida albicans *strains grown in potato dextrose broth medium (PDB). Under aseptic conditions, one mL of spore suspension (5 × 10^6^ cfu/mL) of the fungus being tested was added to 50 mL of PDB medium in a 100 mL Erlenmeyer flask. Appropriate volumes of tested metal complexes were added to produce concentrations ranging from 10 to 100 *μ*g mL^−1^. The flasks were incubated at 27 ± 1°C in the dark for 5 days, at which time the mycelia were collected on filter papers. The filter papers were dried to a constant weight, and the level of inhibition relative to the control flasks was calculated from the following formula:

(1) $$\text{percentage}\;\text{of}\;\text{inhibition}=\frac{C-T}{C}\times 100,$$

where *T* is the weight of mycelia from the test flasks and *C* represents the weight of mycelia from the control flasks.

A note on statistical analysis is that significant differences between values were determined by a multiple-range test (*P* < 0.05) following one-way ANOVA.

#### 2.3.2. Evaluation of Antioxidant Activity

A stock solution (1 mg/mL) was diluted to final concentrations of 20–100 *μ*g/mL. An ethanolic DPPH solution (1 mL, 0.3 mmol) was added to sample solutions in DMSO (3 mL) at various concentrations (50–300 *μ*g/mL) [17]. The mixture was shaken vigorously and allowed to stand at room temperature for 30 min. The absorbance was then measured at 517 nm using the UV-VIS. spectrophotometer. Less absorbance by the reaction mixture indicates higher free-radical-scavenging activity. Ethanol was used as the solvent and ascorbic acid as the standard. The DPPH radical scavenger effect was calculated using the following equation:

(2) $$\text{scavenging}\;\text{effect}\;\left(\%\right)=\frac{A_{0}-A_{1}}{A_{0}}\times 100,$$

where *A*_0_ is the absorbance of the control reaction and *A*_1_ is the absorbance in the presence of the samples or standards.

## 3. Results and Discussion

### 3.1. Chemistry

The ligand was synthesized according to [12]. Reaction could be explained by a Schiff base mechanism. 

**Scheme 1:**
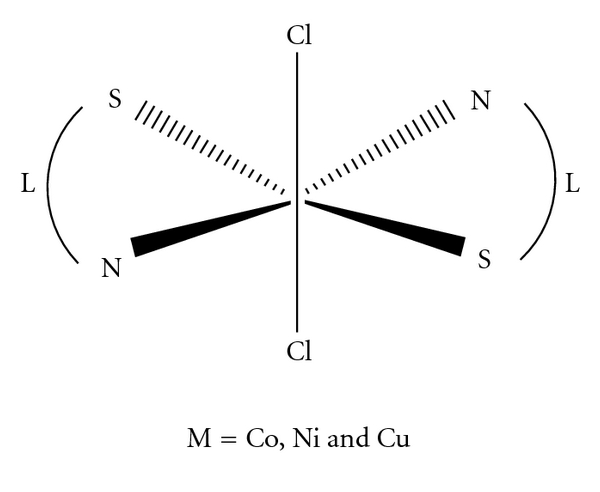
Proposed structure of the complexes.

The complexes (Scheme 1) were then synthesized by the reactions of hot ethanolic solutions of the ligand (L) with the metal ions. The ligand behaves as a bidentate ligand *via* both the thione sulfur and the azomethine nitrogen [12].

#### 3.1.1. Density Functional Theory (DFT)

**Figure 1:**
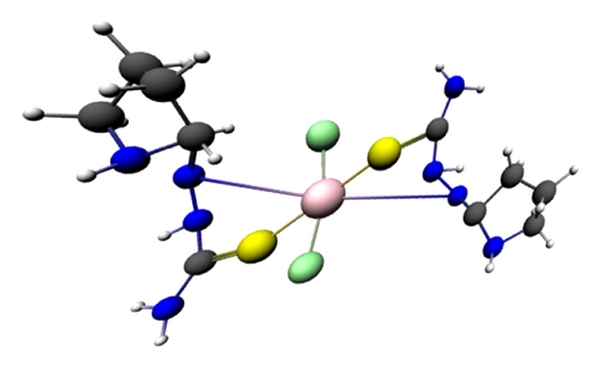
Optimized 3D structure of CuL_2_Cl_2_.

**Figure 2:**
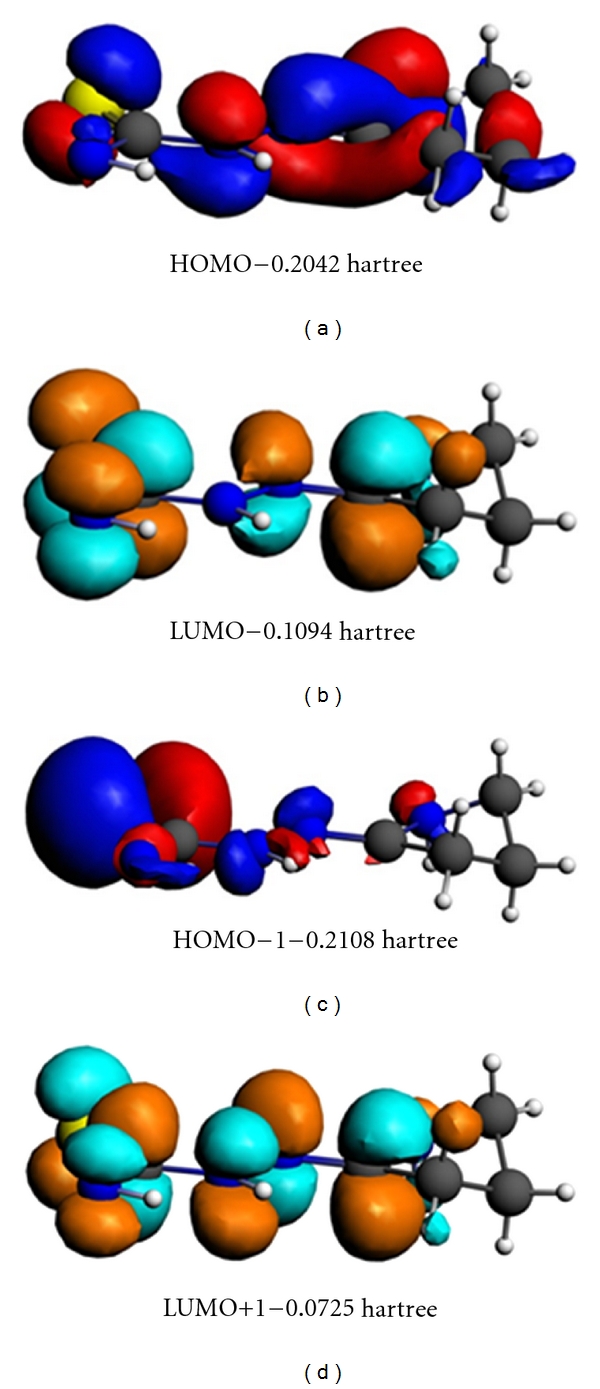
HOMO-LUMO energies for ligand (the energy in hartrees).

DFT calculations were performed for L and CuL_2_Cl_2_. The optimized molecular structure of the most stable form for the Cu complex is shown in Figure 1. Orbital calculations provide a detailed description of the orbitals, including spatial characteristics, nodal patterns, and individual atomic contributions. Contour plots of the frontier orbitals for the ground state of the ligand are shown in Figure 2, including the highest occupied molecular orbital (HOMO) and the lowest unoccupied molecular orbital (LUMO) [18]. It is interesting that both orbitals are substantially distributed over the plane of conjugation. It can be seen from Figure 2 that HOMO orbitals are located on the substituted molecule whereas the LUMO orbitals resemble those obtained for the unsubstituted molecule. Therefore, the substitution has an influence on the electron donation ability but only a small impact on the electron acceptance ability. The orbital energy levels of the HOMO and LUMO for the ligand are listed. It can be seen that the energy gap between the HOMO and LUMO is 0.0984 Hartrees for the ligand. The low value for the HOMO-LUMO energy gap explains the eventual charge transfer interaction taking place within these molecules.

#### 3.1.2. Stereochemistry of the Metal Complexes

**Scheme 2:**
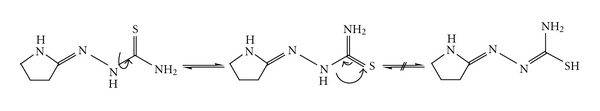


A thiosemicarbazone was first used in this study with the expectation that it would bind to the metal ion as a bidentate N,S-donor. From the preliminary characterization data, it was evident that the thiosemicarbazone ligand does indeed serve as a bidentate ligand, but the coordination mode of the ligand was not clear. The two ligands that are in the coordination sphere around the metal are significantly distorted from the ideal octahedral geometry [19]. To determine the coordination mode of the thiosemicarbazone ligand in these complexes, the structure shows that the thiosemicarbazone ligand is again coordinated to the metal in the same fashion as before. Due to the restricted rotation around the C=N bond, the ligand may exist as two different geometric isomers. The structural determination of one representative ligand (Scheme 2) shows that the free ligand exists in the thione form.

The absence of a thiol group in both the IR and NMR spectra indicates that the ligand exists predominantly as the thione tautomeric form in solution, as shown in Scheme 2. None of the synthesized ligands or metal complexes have any bands between 2,000 and 2,500 cm^−1^, suggesting that the ligand and metal complexes in the solid state are not in the thiol form, as shown in Scheme 2.

### 3.2. Pharmacology

#### 3.2.1. Antifungal Activities

Metal ions are adsorbed on the cell walls of the microorganisms, disturbing the respiration processes of the cells and thus blocking the protein synthesis that is required for further growth of the organisms. Hence, metal ions are essential for the growth-inhibitory effects [20]. According to Overtone's concept of cell permeability, the lipid membrane that surrounds the cell favors the passage of only lipid-soluble materials, so lipophilicity is an important factor controlling the antifungal activity. Upon chelation, the polarity of the metal ion will be reduced due to the overlap of the ligand orbitals and partial sharing of the positive charge of the metal ion with donor groups. In addition, chelation allows for the delocalization of *π*-electrons over the entire chelate ring and enhances the lipophilicity of the complexes. This increased lipophilicity facilitates the penetration of the complexes into lipid membranes, further restricting proliferation of the microorganisms. The variation in the effectiveness of different compounds against different organisms depends either on the impermeability of the microbial cells or on differences in the ribosomes of the cells [21]. All of the metal complexes possess higher antifungal activity than the ligand [22, 23]. Although the exact biochemical mechanism is not completely understood, the mode of action of antimicrobials may involve various targets in the microorganisms. 

These targets include the following.

- The higher activity of the metal complexes may be due to the different properties of the metal ions upon chelation. The polarity of the metal ions will be reduced due to the overlap of the ligand orbitals and partial sharing of the positive charge of the metal ion with donor groups. Thus, chelation enhances the penetration of the complexes into lipid membranes and the blockage of metal binding sites in the enzymes of the microorganisms [24].
- Tweedy's chelation theory predicts that chelation reduces the polarity of the metal atom mainly because of partial sharing of its positive charge with donor groups and possible electron delocalization over the entire ring. This consequently increases the lipophilic character of the chelates, favoring their permeation through the lipid layers of the bacterial membrane [25].
- Interference with the synthesis of cellular walls, causing damage that can lead to altered cell permeability characteristics or disorganized lipoprotein arrangements, ultimately resulting in cell death.
- Deactivation of various cellular enzymes that play a vital role in the metabolic pathways of these microorganisms.
- Denaturation of one or more cellular proteins, causing the normal cellular processes to be impaired.
- Formation of a hydrogen bond through the azomethine group with the active centers of various cellular constituents, resulting in interference with normal cellular processes [26].

**Figure 3:**
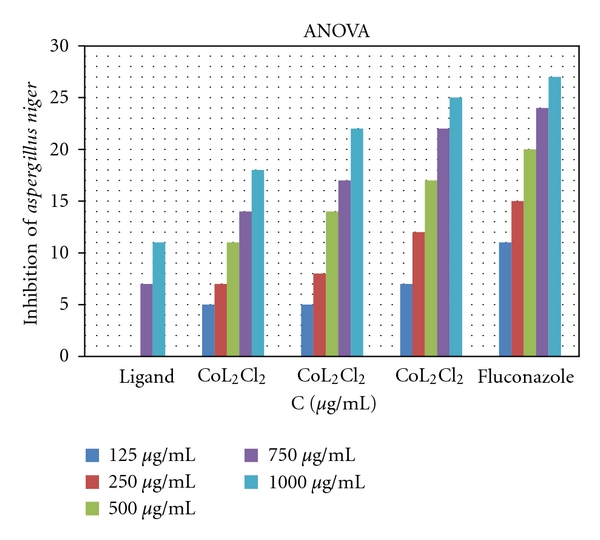
Effect of the metal complexes on *Aspergillus niger*, *P < 0.05, one way Ligand (L) = C_5_H_10_N_4_S; CoL_2_Cl_2 _= Co(C_5_H_10_N_4_S)_2_Cl_2_; NiL_2_Cl_2 _= Ni(C_5_H_10_N_4_S)_2_Cl_2_; CuL_2_Cl_2 _= Cu(C_5_H_10_N_4_S)_2_Cl_2_.

**Figure 4:**
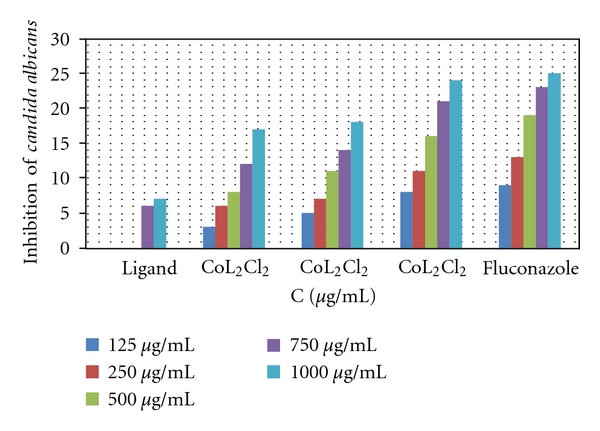
Effect of the metal complexes on *Candida albicans*, *P < 0.05, one-way ANOVA.

*In vitro antifungal* effects of the investigated compounds were tested against two fungal species (*Aspergillus niger* and *Candida albicans*). The results showed that the ligand itself does not exhibit any antifungal activity, but all metal-ligand complexes exhibit good activities. The CuL_2_Cl_2_ shows more activity than NiL_2_Cl_2_ and CoL_2_Cl_2_ which may be due to the higher stability of the CuL_2_Cl_2_ complex (DFT studies, [12], Figures 3 and 4). 

The mode of action of the compounds may involve formation of a hydrogen bond through the azomethine group (>C=N–) with the active centers of various cellular constituents, resulting in interference with normal cellular processes [27, 28]. 

#### 3.2.2. Radical-Scavenging Activity

**Figure 5:**
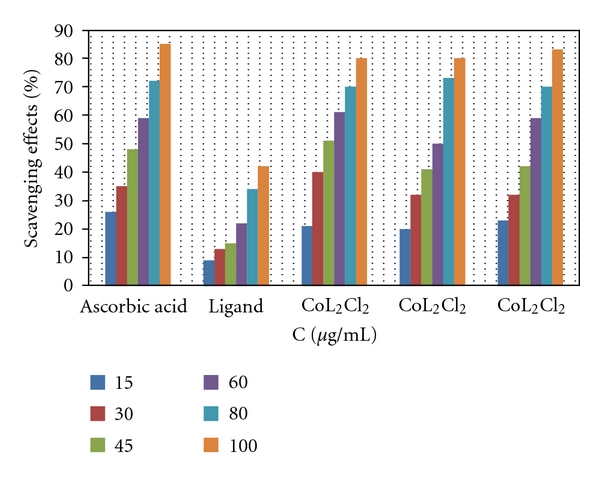
Scavenging effect of metal complexes and ascorbic acid at various concentrations, using the DPPH method.

DPPH is a stable free radical that is often used for detection of the radical-scavenging activity in chemical analysis [29, 30]. The reduction capability of DPPH radicals was determined by the decrease in its absorbance at 517 nm which can be induced by antioxidants. [31]. A graph may be plotted with percentage scavenging effects on the *y*-axis and concentration (*μ*g/mL.) on the *x*-axis. The metal complexes used in the study showed good activities as a radical scavenger compared to the scavenging ability of ascorbic acid, which was used as a standard (Figure 5). These results were in agreement with previous studies of metallic complexes [32, 33] in which the ligand has antioxidant activity and it is expected that the metal moiety will increase its activity.

**Scheme 3:**
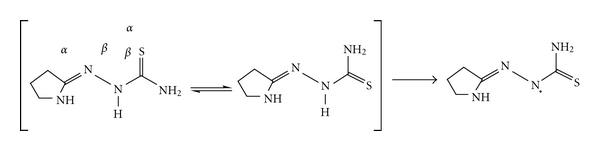
Suggested mechanism for the antioxidant activity of the ligand.

 A postulated mechanism for the antioxidant ability of the ligand is shown in Scheme 3. The mechanism depends on the hydrogen atom of the secondary amine, which is influenced by both the allylic double bond and inductive effects. The allylic stabilization facilitates the release of hydrogen as a free radical, whereas the inductive effect from sulfur and nitrogen pushes electron density toward the free radical, resulting in a relatively stable molecule.

## 4. Conclusions

In this study, a ligand and its metal complexes were tested for antioxidant and antifungal activities. Of the complexes studied, CuL_2_Cl_2_ showed significant antifungal activities compared to either CoL_2_Cl_2_ or NiL_2_Cl_2_. In addition, all complexes were found to be superior antioxidants compared to ascorbic acid. The synthesized compounds were studied theoretically, and the atomic charges, heat of formation, and stereochemistry were estimated. Furthermore, it was found that the compounds are not planar.
